# Strategy Profiles in Solving Algebra Word Problems: A Person-Centered Bayesian Classification Approach for Chinese Students

**DOI:** 10.3390/bs16060953

**Published:** 2026-06-10

**Authors:** Zongzhao Mo, Ronghuan Jiang, Xiaodong Li

**Affiliations:** School of Psychology, Shenzhen University, Nanshan District, Shenzhen 518060, China; 2050042005@email.szu.edu.cn (Z.M.); jrh_psy@szu.edu.cn (R.J.)

**Keywords:** algebra word problems, reversal errors, sentence structures, Chinese language, Bayesian classification approach

## Abstract

This study examined the reversal error phenomenon in Chinese students’ algebra word problem-solving, focusing on how sentence structures influence performance and problem representation strategies across educational levels. Using a person-centered Bayesian classification approach, this study analyzed individual differences in problem-solving strategies across different grade levels. Results confirmed the presence of reversal errors among Chinese students, with sentence structures significantly affecting error rates. Students demonstrated superior performance on congruent problems compared to incongruent problems, showing both fewer errors and faster response times across all grade levels. The study revealed that students processed congruent problems and incongruent problems using fundamentally different strategies. The analysis identified five distinct strategy profiles across grade levels, revealing grade-related differences in strategy use: younger students predominantly relied on direct translation, whereas older students more frequently employed analytic strategies. These findings advance our understanding of cognitive processes in algebra problem-solving and suggest targeted interventions for addressing the reversal error.

## 1. Introduction

Algebra represents a crucial transition from concrete to abstract mathematics. According to the National Council of Teachers of Mathematics (2000), the primary goal of algebra instruction is to enable students to use algebraic language as a tool for representing real-life situations and solving problems. This objective is primarily achieved through algebra word problems. However, research shows that students consistently struggle with these problems (Jupri & Drijvers, 2016; Soneira et al., 2021). A classic example of this difficulty is the “student-professor problem”, which demonstrates the reversal error (RE): “There are six times as many students as professors at this university. Use *S* for the number of students and *P* for the number of professors and write an equation.” In their seminal study, Clement et al. (1981) found that 37% of engineering freshman students failed to solve this problem correctly. The most common mistake was the reversal error, where students wrote “6*S* = *P*” instead of the correct equation “*S* = 6*P*”, thereby inverting the relationship between the variables. The reversal error has proven persistent across diverse groups, including adolescents (Martin & Bassok, 2005; MacGregor & Stacey, 1993), college students (Clement, 1982; Fisher et al., 2011), and even pre-service teachers and high school teachers (Pawley & Cooper, 1997; Soneira et al., 2021).

Cultural factors, language differences, and teaching methods significantly influence students’ mathematical performance (Cai, 2004; Jiang et al., 2017; Soneira et al., 2021). However, research on algebra word problems has been predominantly conducted in Western contexts, with only one notable exception: A study in Hong Kong, which was still conducted in English (Lopez-Real, 1995). This geographical and linguistic limitation is particularly noteworthy given that Chinese, as a logographic rather than phonetic language, may lead to different performance patterns among Chinese students when solving the student-professor problem compared to their Western counterparts. Additionally, previous research (González-Calero et al., 2015; Soneira et al., 2021) has generally analyzed the reversal error as a group phenomenon, potentially obscuring important individual differences in problem-solving approaches and cognitive processes. To address these gaps in the literature, the present study has two primary objectives: (1) Examine how varying sentence structures affect Chinese students’ performance in the student-professor problem. (2) Identify and analyze individual differences in problem-solving strategies using a person-centered approach. Through these objectives, this study aims to enhance our understanding of both cultural and individual factors that influence algebra word problem-solving abilities. The findings could inform the development of more effective teaching strategies and targeted interventions.

### 1.1. The Influence of Sentence Structures on the Reversal Error

Researchers have identified two primary strategies employed by students who make reversal errors: The static comparison strategy and the word order matching strategy (also known as direct translation strategy) (Clement, 1982; González-Calero et al., 2015; Soneira et al., 2021). The static comparison strategy occurs when students treat algebraic letters as labels rather than variables. For example, they might interpret “*S*” and “*P*” as representing “Students” and “Professors” instead of “the number of students” and “the number of professors.” Students using this strategy often misinterpret the equal sign as indicating comparison or association rather than mathematical equivalence. In the student-professor problem, they might interpret the equation “6*S* = *P*” as meaning “six students correspond to one professor.” However, research on these strategies has primarily focused on Western populations, where students regularly use initial letters to represent objects both in educational settings and daily life (e.g., *C* for cats, *D* for dogs). This practice may influence their tendency to treat variable letters as object labels in algebraic problem-solving (Knuth et al., 2005; McNeil et al., 2010; Soneira et al., 2018). The Chinese context offers a distinct perspective. As a logographic rather than phonetic language, Chinese has no inherent relationship between character spelling and pronunciation. Consequently, Chinese speakers rarely use characters as object labels. In Chinese mathematics education, generic letters (*x* and *y*) are the standard choice for representing variables. Our preliminary research with middle and high school students revealed no significant performance differences in the student-professor problem whether variables were represented using English letters, Pinyin (Chinese pronunciation) initials, or generic letters (*x* and *y*). These findings suggest that Chinese students are less likely to employ the static comparison strategy when solving algebraic word problems, including the student-professor problem. Given these observations, the present study will focus exclusively on investigating the direct translation strategy, which appears more relevant to Chinese students’ problem-solving approaches.

The direct translation strategy occurs when students write equations from left to right following the sentence order, leading to incorrect answers in the student-professor problem (Clement et al., 1981; Fisher et al., 2011). The direct translation hypothesis suggests that preventing this strategy could reduce reversal errors. Supporting this theory, González-Calero et al. (2020) found that Basque/Spanish bilingual pre-service teachers made fewer reversal errors when problems were presented in Basque rather than Spanish, as Basque’s linguistic structure naturally inhibits direct translation approaches. Sentence structure has emerged as a significant factor in reversal error occurrence. Soneira et al. (2018) demonstrated that pre-service teachers performed better with statements free from syntactic obstruction (e.g., “The number of students is six times greater than the number of professors”) compared to those with syntactic obstruction (e.g., “There are six students for every professor”). However, the interpretation of such statements can vary significantly across languages. For instance, in Chinese and Danish, the phrase “times greater than” can be semantically different from “times as many as”, potentially leading to a different solution (*S* = (6 + 1) *P* = 7*P*) (Jankvist & Niss, 2021). This linguistic variation means that statements considered unambiguous in one language may become unclear in another.

Notably, research with English-speaking students has produced contrasting results. Christianson et al. (2012) found no significant difference in reversal error rates between problems using helpful phrasing (“The number of students is six times the number of professors”) versus unhelpful phrasing (“There are six times as many students as professors”). These divergent findings across languages and cultures emphasize the importance of further investigating how sentence structure influences reversal errors in different linguistic contexts. In Chinese, two distinct expressions are commonly used for the student-professor problem: (1) “学生人数是教授的6倍” (literally “Students are professors six times”). This is designated as the congruent problem in this study because the direct translation strategy leads to the correct answer (*S* = 6*P*). This Chinese phrasing is more explicit and aligns naturally with the algebraic equation format. Unlike Western students, who show high rates of reversal errors (roughly 40–60%) on this kind of problems (Clement et al., 1981; Soneira et al., 2018), Chinese students may make fewer reversal errors with this phrasing due to its reduced syntactic ambiguity. (2) “每6个学生就有一个教授” (literally “There is one professor for every six students”). This is classified as the incongruent problem because the direct translation strategy results in an incorrect answer. This expression parallels English phrasing structures, and applying the direct translation strategy leads to erroneous solutions. While research has demonstrated that reversal errors persist across age groups (Cohen & Kanim, 2005; González-Calero et al., 2020; Pawley & Cooper, 1997), there remains a significant gap in our understanding of how these errors evolve over time. Few studies have conducted direct comparisons of different age groups within a single study, limiting our understanding of how reversal errors vary across grade levels. This lack of comparative data makes it difficult to assess how students’ problem-solving strategies and their susceptibility to reversal errors may change throughout their educational journey. To address this gap and enhance our understanding of reversal errors in the Chinese educational context, the first aim of this study was twofold: (1) Examine how different surface structures (congruent vs. incongruent phrasing) influenced Chinese students’ algebraic word problem-solving. (2) Investigate how these effects vary across educational levels in algebraic problem-solving strategies. Based on these identified linguistic differences, we propose two hypotheses: (1) Chinese students will demonstrate lower rates of reversal errors on congruent problems compared to their Western counterparts, due to the more explicit phrasing in Chinese. (2) On incongruent problems, Chinese students will show reversal errors rates similar to Western students, as the phrasing parallels English structure and encourages the use of the direct translation strategy (Clement et al., 1981; Kim et al., 2014).

### 1.2. Individual Differences in Strategy Use for Mathematical Problem-Solving

Most prior studies on students’ mathematical problem-solving have analyzed data at the group level (Li et al., 2023; Passolunghi et al., 2022). However, this aggregate methodology may obscure individual differences in the underlying cognitive processes involved. For example, Reinhold et al. (2020) did not find a significant effect of item congruency in fraction comparison tasks for 6th graders when analyzing the whole sample. This was because students with typical bias showed congruency effects in the opposite direction compared to students with reverse bias, causing the group level effect to effectively cancel out. Through cluster analysis, they identified three distinct subgroups: A typical bias cluster, a reverse bias cluster, and a no bias cluster. This exemplifies how person-centered statistical methods are needed to unmask individual differences in strategy use that can be masked by group-level analyses.

While an increasing number of researchers have employed person-centered statistical approaches to unmask individual differences in cognitive processes during mathematical problem-solving, most have utilized explorative techniques such as cluster analysis, latent class analysis, and latent profile analysis (Mo et al., 2026; Reinhold et al., 2020; Vanluydt et al., 2022). However, these explorative person-centered approaches are data-driven, meaning the results greatly rely on and are constrained by the specific sample. This severely impairs the stability and repeatability of the findings. Therefore, to robustly investigate individual differences in strategy use for algebraic word problems, the present study aimed to apply hypothesis-driven (non-explorative) person-centered statistical methods. The naive Bayesian classification approach (the naive Bayes classifier, the NBC) is one of the hypothesis-driven person-centered statistical methods, which is theory-driven and has been used in educational and psychological research in recent years (Culbertson, 2016; Jiang et al., 2026; Leuders & Loibl, 2020; Reinhold et al., 2023). In the naive Bayesian classification approach, all possible strategies that students may use in a certain context are considered as classes, and the features of each class are defined based on psychological theories and previous research findings. These features represent the likelihood of different response patterns within each class. For example, if the features are related to the accuracy of problem-solving, there would be corresponding features indicating the likelihood of achieving certain levels of accuracy on specific problems for each class of strategies. The key assumption in naive Bayesian classification is that the features within each class are independent of each other. With the pre-defined classes and features, the naive Bayesian classification approach analyzes each student’s responses and calculates the posterior probability of each class given the observed responses. Thus, if a student consistently demonstrates a particular response pattern that matches the features of a certain class, the naive Bayesian classification approach will assign a high probability to that class for the student. This process allows for the classification of students based on their observed behavior data.

Recently, some researchers have used the naive Bayesian classification approach to investigate individual differences in strategy use when solving fraction comparison tasks (Reinhold et al., 2023). They first identified the classes of comparison strategies based on the conceptual change account (Vamvakoussi & Vosniadou, 2004) and the dual process account (Dooren & Inglis, 2015). For each class, there is a feature that described the likelihood of the accuracy and response time to different types of fraction problems. Students were then classified by the naive Bayesian classification approach. The results not only corroborated existing strategy patterns identified in previous studies but also revealed additional composite strategy patterns. This hypothesis-driven approach helps to reduce misjudgments stemming from subjective factors and improves classification accuracy compared to exploratory clustering methods. Researchers have employed various methods to investigate the cognitive processes underlying the student-professor problem, with clinical interviews emerging as a valuable tool for revealing individual differences in problem-solving strategies (Adu-Gyamfi et al., 2015; Clement, 1982; Wollman, 1983). In a notable study, Adu-Gyamfi et al. (2015) conducted clinical interviews with college students to examine their cognitive processes during problem-solving. Their findings identified several distinct types of translation errors, including errors in attribute construction, implementation construction, equivalence construction, and unclear construction. However, these interview-based studies on strategy use tend to have limited sample sizes, reducing the representativeness and generalizability of the strategy classification results. Therefore, to comprehensively examine individual differences in cognitive processes underlying the student-professor problem, we proposed a more complete spectrum of hypothesized strategy use patterns based on potential underlying cognitive mechanisms. We then empirically tested the existence of these hypothesized strategy patterns through the naive Bayesian classification approach.

### 1.3. Cognitive Mechanisms Underlying Students’ Strategy Use

Our study examines student strategies in solving the student-professor problem through the lens of dual-process theory and the inhibitory control model. Dual-process theory (Evans, 2003; Evans & Stanovich, 2013) proposes two distinct cognitive processing systems: The heuristic system (S1) which is fast, effortless, and automatic and the analytic system (S2), which is slow, effortful, and demanding. While heuristic processes are typically adaptive and activate first, the analytic system must intervene when heuristic solutions conflict with correct outcomes. In algebra word problems, researchers have linked reversal errors to a left-right direct-translation heuristic (Fisher et al., 2011; González-Calero et al., 2020). Based on dual-process theory, we propose that the direct translation strategy emerges from the heuristic system, automatically activating when students encounter student-professor problems. This heuristic response likely stems from two factors: (1) The structural similarity between algebraic equations and sequential linear relational statements (Fisher et al., 2011). (2) Students’ repeated success using direct translation in solving mathematical word problems (Hegarty et al., 1995; Lubin et al., 2016; Passolunghi et al., 2022).

The effectiveness of these strategies varies by problem type: In congruent problems, direct translation (heuristic system) leads to correct equations and fast responses. In incongruent problems, direct translation yields incorrect solutions and fast responses, requiring students to employ the analytic strategy (analytic system) to achieve correct but slower responses.

While dual-process theory explains the coexistence of heuristic and analytical processes, it doesn’t fully account for how students successfully transition from heuristic to analytical approaches when solving incongruent problems. The inhibitory control model (Houdé & Borst, 2014, 2015) addresses this gap by emphasizing the critical role of inhibitory control mechanism. According to this model, misleading heuristics and correct strategies co-exist in the brain throughout development, but since heuristics are prepotent, students must engage inhibitory control to overcome them and reach correct solutions. Research has consistently demonstrated the importance of inhibitory control in this process (Jiang et al., 2019; Li et al., 2023). The inhibitory control model suggests two potential reasons for problem-solving failures (Jiang et al., 2020; Mevel et al., 2014): (1) Lack of conflict detection: Students fail to recognize the mismatch between the misleading strategy and the problem situation. (2) Insufficient inhibition: Students detect the conflict but cannot successfully inhibit the misleading strategy. This creates a hierarchical process where conflict detection serves as prerequisite for inhibitory control. Without conflict detection, there is no trigger for inhibitory mechanisms. Even with successful conflict detection, students may still apply the misleading heuristic if they cannot effectively inhibit it.

Based on this framework, we posit that successful resolution of the student-professor problem depends on two key mechanisms: (1) Conflict detection: Recognizing when direct translation strategies are inappropriate. (2) Inhibitory control: Successfully suppressing the misleading direct translation heuristic. The interaction of these mechanisms can create distinctive patterns in both problem-solving accuracy and response times. Inaccurate responses typically stem from two potential failures: Either a breakdown in conflict detection (such as failing to recognize contextual incompatibility) or a lapse in inhibitory control (continuing with the heuristic approach despite recognizing conflicts). To achieve accurate responses, both mechanisms must function successfully. Response times provide additional insight into these cognitive processes. Studies across various tasks (Babai et al., 2012; Frey et al., 2018; Jiang et al., 2020; Pennycook et al., 2012; Stupple et al., 2013) indicate that conflict detection requires more time than non-conflict scenarios, as it involves evaluating contextual ambiguity. Additionally, inhibitory processes—when suppressing a dominant but incorrect strategy—lead to longer response times compared to situations not requiring inhibition. Therefore, extended response times may indicate the engagement of both conflict resolution and inhibitory mechanisms.

In the present study, we propose four distinct profiles of students solving the student-professor problem. These profiles are: Automatic Translators, Conflict Recognizers, Strategic Suppressors, and Conceptual Integrators. Automatic Translators fail to detect conflicts in incongruent problems, applying direct translation indiscriminately. These students correctly solve congruent problems but make reversal errors on incongruent ones, displaying uniformly fast response times across both problem types. Conflict Recognizers recognize when direct translation conflicts with incongruent problems but fail to inhibit this inappropriate strategy despite detecting the conflict. Like Automatic Translators, they solve congruent problems correctly but make reversal errors on incongruent ones. However, they respond more slowly to incongruent problems due to the additional cognitive processing required for conflict detection. Strategic Suppressors demonstrate more sophisticated problem-solving abilities. They appropriately use direct translation for congruent problems while successfully inhibiting this strategy for incongruent problems in favor of an analytic approach. These students answer both problem types correctly but work more quickly on congruent problems. Conceptual Integrators represent the most advanced profile, handling both problem types with an efficient approach. They demonstrate consistently correct answers with similar response times across problem types, indicating genuine conceptual understanding (Reinhold et al., 2023). Beyond these four main profiles, some students may be classified as Random Guessers, responding arbitrarily to all problems regardless of type (Reinhold et al., 2023).

### 1.4. The Present Study

This study had two primary objectives: (a) To investigate how different sentence structures influenced Chinese students’ performance and problem representation strategies on the student-professor problem across different educational levels, particularly examining the congruency effects and testing the heuristic explanation; and (b) to profile individual differences in cognitive patterns of problem-solving using a person-centered Bayesian classification approach, thereby identifying distinct strategy profiles among students across different grade levels. To address these aims, we developed a two-alternative forced-choice (2AFC) computerized task comprising congruent and incongruent student-professor problems and recruited participants from middle school to college levels. Notably, the present study considered students’ behavioral data (accuracy and response time) simultaneously when defining the features for each class, in contrast to Reinhold et al.’s (2023) approach, in which the two response types were analyzed separately. This simultaneous approach yields more accurate and detailed classifications, as accuracy and response time are jointly generated by a single response rather than arising from two separate responses (Jiang et al., 2026). In a single-response event, accuracy and RT are not independent outputs of separate cognitive modules; rather, they are joint observable signatures of a unified underlying process—most commonly formalized as evidence accumulation. When a participant makes a single categorization decision, the same evidence accumulation dynamics that determine whether the response is correct (accuracy) also govern how long the process takes (RT). Treating them separately, as in the two-stage approach by Reinhold et al. (2023), imposes an artificial decomposition: it assumes that the cognitive information relevant to accuracy is fully captured in the first stage, leaving RT as a residual explained only by secondary factors. This misrepresents the cognitive reality in which parameters such as drift rate, boundary separation, and non-decision time simultaneously shape both accuracy and RT. We hypothesized the following: (1) Students would interpret congruent and incongruent problems as distinct problem types; (2) Sentence structures would affect reversal errors, such that students would perform better and respond faster on congruent problems compared to incongruent problems, regardless of grade level; (3) Individual differences in strategy use patterns would be found, and such individual differences would vary from grade levels.

## 2. Method

### 2.1. Participants

We used G*Power 3.1 (Faul et al., 2007) to calculate the sample size. The calculation yielded a total sample size of 76–192 (with 19–48 participants in each grade) with an effect size of 0.15–0.25 and a statistical power of 95%. To account for potential data loss, we recruited 49 seventh-grade students (27 boys, 22 girls; mean age: 13.39 ± 0.53 years), 52 eighth-grade students (27 boys, 25 girls; mean age: 13.57 ± 0.54 years) and 58 eleventh-grade students (42 boys, 16 girls; mean age: 17.22 ± 0.56 years) from a middle school in Zhaoqing, China. For young adults, we recruited 54 science and engineering (calculus-level) undergraduates or graduates (28 male, 26 female; mean age: 20.83 ± 1.89 years) from a university in Shenzhen, China. They received extra course credit for their participation. All participants reported normal or corrected-to-normal vision, had never participated in a similar experiment before, provided informed consent (or a guardian’s informed consent), and were tested in accordance with the national and international norms of using human participants. The present study was approved by the research ethics committee of the Shenzhen University.

### 2.2. Materials

In our study, there were 16 problems, with 8 congruent problems and 8 incongruent problems. For congruent problems, the direct translation strategy contributed to correct answers. However, correct solutions for incongruent problems conflicted with the direct translation strategy. The critical sentence of congruent problems was expressed as “The number of *X* times n is m times the number of *Y*”, while for incongruent problems it was “There are n *X* for every m *Y*”, where n and m were mutually prime integers ranging from 1 to 9 and *X* and *Y* were two categorically related objects (e.g., apples and pears). Note that when n was 1, congruent problems can be expressed as “The number of *X* is m times the number of *Y*.” Likewise, when m was 1, incongruent problems can be articulated as “There are n *X* for every *Y*.” Moreover, following previous studies (González-Calero et al., 2015; Kim et al., 2014; Soneira et al., 2018), we controlled for types of magnitudes (using discrete variables rather than continuous variables) and contextual clues (not suggesting which quantity was greater).

As shown in Table 1, in this study we developed a two-alternative, forced-choice (2AFC) computerized task. Specifically, each item provided two equations, one correctly represented the relational statement, the other was an inappropriate reverse representation. Participants were asked to select the correct equation for the student-professor problem. To validate the study instruments, we conducted pilot testing with twelve participants: Six eighth-grade students and six college students majoring in science and engineering, with equal gender representation in each group. Based on their feedback, we refined the items before administering them to the full study sample.

### 2.3. Procedure

Middle school students and high school students were tested in multimedia classrooms equipped with computers. As for college students, they performed the task individually in a lab. All participants were seated approximately 60 cm in front of the computers. The stimuli were presented with a screen resolution of 1280 × 768 pixels, using the E-Prime 3.0 software (Psychology Software Tools, Pittsburgh, PA, USA). To familiarize themselves with the tasks, participants were first asked to perform two practice trials without feedback, including one congruent problem and one incongruent problem. These trials were presented randomly and were not used in the formal experiment.

**Figure 1:**
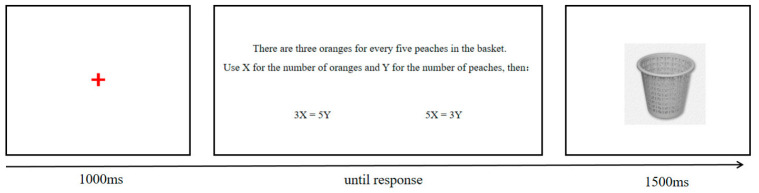
Procedure of experimental trials.

After completing practice trials, participants performed 16 experimental trials and the procedure was shown in Figure 1. Participants were requested to select the equation that correctly represented quantitative relationship as quickly and accurately as possible by pressing the “*Q*” key to choose the left equation or the “*P*” key to choose the right equation. Correct equations were equally distributed on the left and right sides across congruent and incongruent problems. To minimize practice effects and response patterns, we presented the experimental trials in a pseudorandom sequence, ensuring that no more than two congruent or incongruent problems appeared consecutively more than twice successively. Response times (RTs) and error rates (ERs) for all experimental trials were recorded.

### 2.4. Data Analysis

One participant’s data was lost during the experiment, leaving 212 students for the Bayesian classification analysis. A further two participants were excluded from the mean error rate and response time analyses because their RTs fell below 200 ms in more than 50% of trials, which may distort descriptive statistics for group-level performance (Simpson & Todd, 2017). These two participants were nonetheless retained in the Bayesian classification analysis, as this approach operates at the individual level and includes a hypothesized class of students who may adopt a random guessing strategy. At the trial level, RTs below 200 ms were removed, and RT outliers exceeding three standard deviations above or below each participant’s mean RT were also excluded (Simpson & Todd, 2017; Todd & Simpson, 2016).

Firstly, to examine how participants represented the two types of student-professor problems (congruent and incongruent), we conducted a confirmatory factor analysis (CFA) with Weighted Least Squares Mean and Variance adjusted estimation (WLSMV) using Mplus 7.0 (Muthén & Muthén, 2012). Next, we calculated the mean ERs and RTs separately for congruent and incongruent problems for each participant, and defined students’ responses for each problem by combining their accuracy and RT. Specifically, a student’s actual response to each item was defined as: Correct-faster, correct-slower, incorrect-faster, incorrect-slower, based on their accuracy and RT relative to the median RT for all problems. To determine the faster/slower categorization, we first normalized RTs by using the median RT as the reference point. Normalized RTs greater than 0 were classified as “slower”, and those less than 0 were classified as “faster”. Finally, we classified the students into distinct groups based on their response patterns (accuracy and response times) using a Bayesian classification approach.

The naive Bayesian classification approach assumes that all attributes (the evidence *X*) of the test samples (students) are mutually independent (Duda et al., 2012). Consequently, one student’s (e.g., the student *k*) posterior probabilities of all strategies can be obtained from the following formula:

(1) $$P_{k}\left(C_{i}|X\right)=\frac{P_{k}(X|C_{i})P_{k}(C_{i})}{P_{k}(X)}=\frac{P_{k}(C_{i})}{P_{k}(X)}\prod_{j=1}^{16}P_{k}\left(X_{j}|C_{i}\right)\;(1\leq j\leq 16,\;1\leq i\leq 8)$$

Note that $P_{k}(C_{i}|X)$ expresses posterior probabilities of the student *k* using the strategy $C_{i}$, $P_{k}(X)$ expresses the probability of the evidence *X* (i.e., response of the student *k* to each item), $P_{k}(X|C_{i})$ represented the conditional probability of the evidence *X* under the condition of the student *k* using the strategy *C_i_*, $P_{k}(C_{i})$ expresses the probability of the student *k* using the strategy *C_i_*. And then the student *k* is divided into the strategy based on the Bayes factors (BFs, probability ratios) from the following formula:

(2) $$\frac{P_{k}(C_{i}|X_{m})}{P_{k}(C_{i+1}|X_{m})}=\prod_{m=1}^{16}\frac{P_{k}\left(X_{m}|C_{i}\right)}{P_{k}\left(X_{m}|C_{i+1}\right)}*\frac{P_{k}(C_{i})}{P_{k}(C_{i+1})}\;(1\leq m\leq 16,\;2\leq i+1\leq 9)$$

According to Formula (1), we proposed a probability distribution for all strategies student *k* might use (i.e., *P_k_*(*C_i_*), we assigned 0.2 as an initial prior probability for every strategy since there were five patterns). This probability was then updated with each piece of evidence *X* (i.e., each student’s response to each item). Since the likelihoods for evidence *X* (i.e., $P_{k}(X|C_{i})$) had no exact probabilities, according to prior literature (Reinhold et al., 2023), we assigned 0.03 as a low probability, 0.5 as a medium (chance) probability, and 0.91 as a high probability for evidence *X* under the condition of student *k* using strategy *C_i_* (see Table 2). For example, the probability of correctly (and faster) solving a congruent problem was 0.91 for student *k* using the direct translation strategy, while the likelihood was 0.03 for student *k* successfully (and faster) finishing an incongruent item using the direct translation strategy. The likelihood was 0.25 for the guessing strategy.

**Figure 2:**
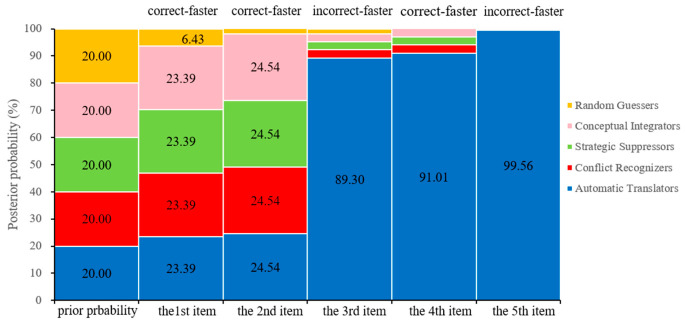
One example of naive Bayesian classification approach on students’ response. *Note*. Congruent problems: the 1st item, 2nd item, and 4th item; incongruent problems: the 3rd item and 5th item.

From Formula (1), we can see that if student *k* responded consistently to all (or most) items using one strategy, then cumulative evidence would yield a considerably increasing posterior probability for this strategy via Bayesian updating. For example, consider the order of student *k*’s responses on the first five student-professor problems with different responses (see Figure 2). For the first problem, the likelihood was high (0.91) for Automatic Translators, Conflict Recognizers, Strategic Suppressors, and Conceptual Integrators. Using Formula (1), the posterior probabilities for these four strategies were 0.23.^1^ The likelihood was medium (0.25) for Random Guessers, yielding a posterior probability of 0.06.^2^ Subsequently, the posterior probabilities from the first problem became the prior probabilities for the second problem, which were then updated based on the evidence from the second problem. After completing five items, the posterior probability of student *k* for Automatic Translators had accumulated to more than 99%. After finishing all 16 items, we obtained the final posterior probabilities.

Student *k* should be grouped based on the Bayes factors (probability ratios) from the Formula (2). We classified student *k* into the class of the strategy with the highest posterior probability if the Bayes factor of the two dominant strategies (the two strategies with the highest posterior probabilities) was greater than 3 (Jeffreys, 1961; Wagenmakers et al., 2018). For example, if student *k* had the highest posterior probability of 0.80 for Automatic Translators, and the second highest posterior probability of 0.10 for Conflict Recognizers, then the Bayes factor would be 0.80/0.10 = 8 and student *k* would be classified into the “Automatic Translators” class. If the Bayes factor of the two dominant strategies was less than 3 (Jeffreys, 1961; Wagenmakers et al., 2018), student *k* would be classified into the “Mixed Strategies Users” class.

## 3. Results

### 3.1. Result of a Confirmatory Factor Analysis

To evaluate the model fit, we applied the following criteria: the RMSEA should be less than 0.08, the CFI and TLI should be greater than 0.90, the *χ*^2^/*df* should be less than 3, and the WRMR should be less than 1 (Muthén & Muthén, 2012). Fit-comparison statistics further indicated that ΔCFI = 0.085 and ΔRMSEA = 0.137, both exceeding the recommended cutoffs for meaningful model improvement (Chen, 2007). As shown in Table 3, the two-factor model demonstrated superior fit relative to the one-factor model. All items loaded strongly on their respective factors, and the two factors were negatively correlated, supporting discriminant validity (see Appendix A Table A1). Together, these findings suggest that students perceived congruent and incongruent problems as qualitatively distinct problem types when solving the student-professor problems.

### 3.2. Descriptive Results

Descriptive results are shown in Table 4.

#### 3.2.1. ERs

**Figure 3:**
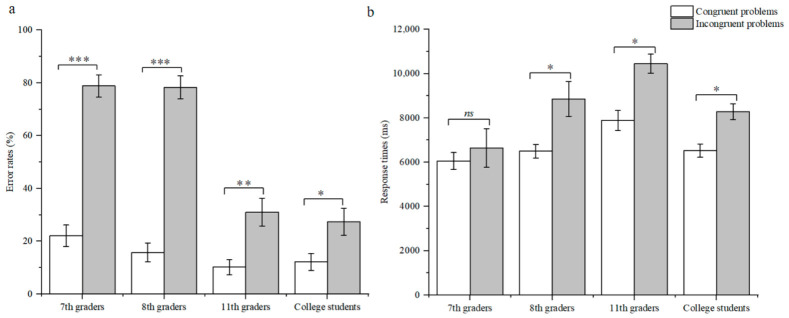
Error rates (**a**) and response times (**b**) for students. Error bars mean the standard error of the mean. *ns =* non-significant; * *p* < 0.05; ** *p* < 0.01; *** *p* < 0.001.

Shapiro–Wilk tests revealed that ERs violated the normal distribution for each group and each type of problem (*p*s < 0.01), thus we conducted a Wilcoxon signed-rank test. We found that participants performed better in congruent problems than incongruent problems at every grade level (see Figure 3). A Kruskal–Wallis test revealed that students’ grades were significantly related to their performance on congruent problems, *χ*^2^(3) = 8.16, *p* = 0.043, and incongruent problems, *χ*^2^(3) = 64.49, *p* < 0.001. For congruent problems, 7th graders performed worse than 11th graders and college students. However, there was no difference between 7th graders and 8th graders in the reversal error on those problems. No difference in performance was found among other grade levels. In contrast, for incongruent problems, both 7th graders and 8th graders committed more reversal errors than 11th graders and college students, while 11th graders and college students exhibited no difference in the reversal error on these problems. No significant differences in ERs were found between male and female students across all grader levels (see Appendix A Table A2).

#### 3.2.2. RTs

Shapiro–Wilk tests confirmed that RTs were normally distributed for each group and problem type (*p*s > 0.05); thus we conducted a 2 (sentence structures: congruent problems, incongruent problems) × 4 (grade levels: 7th grade, 8th grade, 11th grade, college students) repeated measures ANOVA. We found a significant main effect of sentence structures, *F* (1, 209) = 27.33, *p* < 0.001, *η*^2^ = 0.17, with longer RTs for incongruent compared to congruent problems. The main effect of grades was also significant, *F* (3, 207) = 9.26, *p* < 0.001, *η*^2^ = 0.17. The Bonferroni post hoc test showed that 11th graders responded slower than the other grade levels, while 7th graders, 8th graders and college students did not differ between grade levels. The interaction between grades and sentence structures was not significant, *F* (3, 207) = 0.55, *p* = 0.65, *η*^2^ = 0.012. Further simple effect analysis suggested that 7th graders exhibited no difference between congruent and incongruent problems. However, 8th graders, 11th graders, and college students all had shorter RTs for congruent problems versus incongruent problems, *p* = 0.04, *p* < 0.001, *p* = 0.001, respectively (see Figure 3). No significant differences in RTs were found between male and female students across all grade levels (see Appendix A Table A3).

### 3.3. Individual Differences in Strategy Use

**Figure 4:**
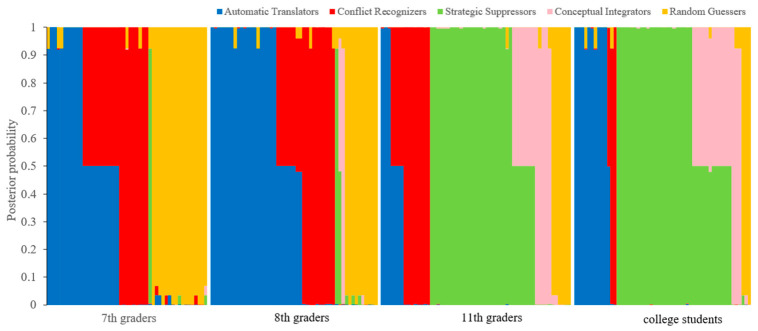
Posterior probabilities of five distinct profiles of students in different grades.

**Figure 5:**
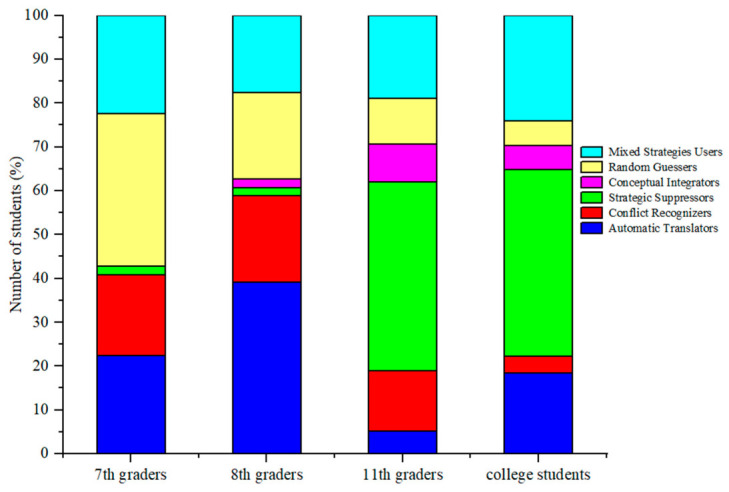
Number of students (%) classified into the hypothesized strategies in every grade.

The Bayesian classification analysis revealed five distinct student profiles across all grade levels when solving the student-professor problem (see Figure 4), supporting our theoretical assumptions. However, the distribution of these profiles varied significantly by grade level. A chi-square test confirmed these grade-level differences in strategy use, *χ*^2^ (15, *N* = 212) = 79.92, *p* < 0.001. Among 7th and 8th graders, the highest posterior probabilities were observed for three profiles: Automatic Translators, Conflict Recognizers, and Random Guessers. In contrast, 11th graders and college students showed the highest posterior probabilities for Strategic Suppressors. When solving incongruent problems specifically, 7th and 8th graders relied more heavily on direct translation strategies (as Automatic Translators and Conflict Recognizers) and guessing strategies (as Random Guessers) compared to older students (see Figure 5). Conversely, 11th graders and college students more frequently employed the analytic strategy (as Strategic Suppressors) for incongruent problems. Notably, some students across all grade levels demonstrated highest posterior probabilities for multiple profiles, indicating the use of “mixed” strategies when approaching congruent or incongruent problems. These students were classified as Mixed Strategies Users. The complete distribution of students across all strategies and grades is presented in Table 5. Furthermore, we conducted a sensitivity analysis by systematically varying the three likelihood values across plausible ranges (e.g., 0.05/0.50/0.85). The results, presented in Appendix A Table A4, demonstrate that the main strategy profiles and classification outcomes remain qualitatively stable across these variations.

To further investigate the idiosyncratic strategy use of patterns of the Mixed Strategies Users group, we classified these individuals based on their dynamic shifts in strategy use across the 16-item task (see Table 6). Contrary to constituting a homogeneous group with consistent strategy use patterns, Mixed Strategies Users could be parsed into four qualitatively distinct transition profiles.

First, a subset of students exhibited the Automatic Translator to Conflict Recognizer profile (15.38% in Grade 7, 11.11% in Grade 8, and 27.27% in Grade 11). This pattern reflects a qualitative shift from the heuristic-based processing—characterized by correct-fast responses on congruent problems and incorrect-fast responses on incongruent problems—toward the conscious detection of response conflict, typically manifested as a deceleration in response times as cognitive control mechanisms engage, resulting in incorrect-slow responses on incongruent items.

Second, the Cautious Solver to Automatic Translator profile was the most prevalent among younger students (53.85% in Grade 7 and 88.89% in Grade 8), with markedly lower representation in Grade 11 (9.09%) and no occurrence among college students. Students in this profile initially employed controlled, deliberate processing on congruent problems but gradually shifted toward automatic application of the direct translation strategy, while persistently producing errors on incongruent items.

Third, the Strategic Suppressor to Conceptual Integrator profile predominated among older students (63.64% in Grade 11 and 69.23% among college students). This profile is characterized by an initial capacity to suppress the incorrect translation strategy on incongruent problems, which gradually gives way to efficient, schema-based processing that supports accurate performance on both congruent and incongruent problem types, reflected in correct-fast responses across both conditions.

Finally, a minority of students displayed the Genuinely Idiosyncratic Solver profile, observed exclusively among college students (30.77%) and one 7th-grade student (7.69%). This pattern is characterized by unsystematic fluctuations in strategy use across items, indicating the absence of a stable, rule-based response strategy and suggesting highly individualized approaches to problem solving that do not conform to any identifiable developmental trajectory.

## 4. Discussion

The present study had two primary aims: (1) to examine how sentence structure affected Chinese students’ performance and problem representation on the student-professor problem across educational levels; and (2) to identify individual differences in problem-solving strategies across grade level.

Our findings revealed that students across all grade levels perceived and processed congruent problems (e.g., “Students are professors six times”) and incongruent problems (e.g., “There are six students for every professor”) as fundamentally different. Consistently, students demonstrated both higher accuracy and faster response times on congruent problems compared to incongruent ones.

Most notably, we identified five distinct strategy profiles among students. Clear grade-related differences emerged: middle school students predominantly relied on direct translation strategies, whereas high school and college students demonstrated greater strategic flexibility. Specifically, these older students adapted their approach based on sentence structure, employing direct translation for congruent problems and shifting to analytic strategy for incongruent problems.

The confirmatory factor analysis revealed that Chinese students perceived congruent and incongruent problems as distinct types, despite both containing the same quantitative relationships. This finding indicates that students’ problem representations were influenced by sentence structures, suggesting they approached word problems based on surface structure (syntactic translation) rather than deep structure (quantitative relationship).

Despite recognizing these problems as distinct types, many students nonetheless attempted to apply direct translation universally, resulting in reversal errors on incongruent problems. This supports our hypothesis that sentence structure contributes to reversal errors in the student-professor problem. Notably, although Chinese syntax produced performance differences on congruent problems relative to Western peers (Fisher et al., 2011; González-Calero et al., 2020), Chinese students exhibited reversal errors on incongruent problems at rates comparable to those of their Western counterparts (Clement et al., 1981; Kim et al., 2014). We caution, however, against direct numerical comparison between our 2AFC error rates and those reported in production-task studies (Clement et al., 1981; Soneira et al., 2021). We acknowledge that our 2AFC recognition task likely yields a lower-bound estimate of reversal error prevalence and an overestimate of students’ true algebraic competence, because the recognition format reduces cognitive load in two ways: the presented alternatives provide additional cues that reduce working memory demands, and students are not required to generate a complete equation independently, thereby reducing executive processing costs. Contrary to this interpretation, Mo et al. (2026) provided validation evidence that Chinese eighth graders made fewer reversal errors on the production-task than participants in the present study who completed the 2AFC recognition task, for both congruent problems (11.44% vs. 15.74%) and incongruent problems (36.82% vs. 78.32%). This finding suggests that the 2AFC recognition format does not necessarily improve equation-solving performance.

Grade-level analysis revealed substantial differences in reversal error rates: 7th graders (78.83%) and 8th graders (78.32%) made considerably more errors than high school students (31.07%) and college students (27.39%). Given that algebraic equation concepts are introduced in Chinese 5th and 7th grades, these error patterns cannot be attributed solely to insufficient conceptual knowledge. Rather, we propose that these grade-related differences reflect variations in students’ inhibitory control abilities (Jiang & Li, 2017; Jiang et al., 2019; Lubin et al., 2016). While the direct translation strategy successfully solves congruent problems, incongruent problems require inhibiting this strategy—a process that imposes a cognitive cost reflected in longer response times. Supporting this interpretation, 11th graders and college students showed longer response times on incongruent versus congruent problems, suggesting successful inhibition of the direct translation strategy. In contrast, 7th graders showed no response time differences between problem types, indicating failure to inhibit the direct translation strategy. Because the present study did not include 9th and 10th graders, a complete developmental trajectory could not be established; future research should address this gap by incorporating a continuous grade-level sample.

The Bayesian classification analysis revealed distinct patterns of strategy use across grade levels. Among 7th and 8th graders, the predominant approach was direct translation for both congruent and incongruent problems, manifesting in two profiles: Automatic Translators and Conflict Recognizers. Automatic Translators failed to detect the differences between problem types, while Conflict Recognizers recognized the conflict between the direct translation and incongruent problems but failed to successfully inhibit this inappropriate strategy, consistent with the inhibitory control model. Notably, successful inhibition of the direct translation strategy on incongruent problems was extremely rare among middle school students with only one student each from 7th grade and 8th grade classified as Strategic Suppressors. In contrast, 11th graders and college students demonstrated markedly different strategy patterns. The Bayesian classification indicated that most of these older students successfully inhibited the direct translation strategy when solving incongruent problems. As Strategic Suppressors, they correctly solved both problem types by suppressing the misleading strategy when necessary.

The data revealed an intriguing pattern: Some students failed even on congruent problems (22.06% of 7th graders and 15.74% of 8th graders), despite presumably using the direct translation strategy. We propose this performance deficit stems from incomplete conceptual understanding of algebra. This interpretation is strengthened by the Bayesian classification results, which identified a substantial proportion of students who appeared to be guessing randomly on student-professor problems (Random Guessers: 34.69% of 7th graders and 19.61% of 8th graders).

Consistent with findings from Western samples, Chinese students similarly exhibited reversal errors on incongruent problems. Our analysis also identified a “mixed” group whose strategy use could not be classified into a single profile, suggesting that these students employed varied and flexible problem solving approaches—a pattern consistent with overlapping waves theory (Opfer & Siegler, 2007; Siegler, 1998). According to this theory, cognitive development involves increasing variability and flexibility in strategy use prior to consolidation into dominant approaches.

The Bayesian classification approach revealed important nuances that would have been obscured by aggregate analyses alone. By identifying distinct subgroups with various error patterns and strategy preferences, this fine-grained analysis provides a more complete understanding of the diverse reasoning profiles that underlie students’ performance on these problems.

This study, while offering valuable insights into the cognitive processes of algebraic word problem solving across grade levels, has several limitations. A fundamental challenge lies in inferring cognitive processes from behavioral data alone (accuracy and response times), as there remains an inherent gap between observable performance and underlying thought processes. Future research would benefit from a mixed-methods approach, combining quantitative measures like paper-pencil tests and eye tracking with qualitative methods such as clinical interviews, to build a more comprehensive understanding of students’ problem-solving cognition.

While our Bayesian classification approach successfully identified subgroups showing distinct response time patterns between congruent and incongruent problems, our interpretations of these patterns require further validation. We proposed that inhibitory control mechanisms underlie students’ ability to overcome the misleading direct translation strategy, with longer response times potentially reflecting this inhibition process. However, direct empirical evidence is needed to confirm whether slower or less accurate performance truly reflects an inhibition mechanism. Future studies could employ specialized methodologies, such as the Negative Priming paradigm, to specifically investigate the role of inhibitory control in overcoming the intuitive direct translation strategy when solving incongruent algebra word problems. Furthermore, although the naïve Bayesian classification analysis is hypothesis-driven, the distribution of the identified subgroups may be affected by a systematic sampling bias. For example, in the present study, the 11th-grade sample comprised substantially more boys than girls, and prior research has suggested that gender differences exist in inhibitory control and mathematical problem-solving. Such gender imbalance may influence the proportion of students assigned to different subgroups. Future studies should therefore aim for balanced gender representation across grade-level samples.

Finally, our findings have significant implications for algebra instruction. First, teachers should recognize the prevalence and persistence of reversal errors in students’ solutions to algebraic word problems. These errors often arise not solely from a lack of conceptual understanding but from the misapplication of intuitive translation strategies. When linguistic structures mislead them, students must inhibit these strategies to succeed. Solving such problems requires not only relevant knowledge and skills, but also the inhibitory control to override misleading heuristics or overlearned strategies. Teachers must be aware of this cognitive demand.

Second, the study underscores the importance of teachers gaining deeper insights into the diverse cognitive processes and strategy profiles students employ. For instance, slower problem-solving on incongruent problems may reflect inhibition demands, while direct guessing often signals gaps in foundational knowledge. A uniform instructional approach is unlikely to address these varied challenges effectively. Instead, curricula should focus on helping students recognize conflicts between linguistic and quantitative problem structures and strategically inhibit misleading heuristics when necessary. Such an approach could enhance algebraic reasoning instruction and address the persistent reversal errors. By understanding the range of strategies, error patterns, and cognitive demands students encounter, teachers can provide more targeted and effective support.

## Figures and Tables

**Table 1:** Examples of congruent problems and incongruent problems.

Congruent Problems		Incongruent Problems	
In the classroom, the number of boys is four times the number of girls. Use *X* for the number of boys and *Y* for the number of girls, then:		In the zoo, there are five tigers for every lion. Use *X* for the number of tigers and *Y* for the number of lions, then:	
4*X* = *Y*	*X* = 4*Y*	*X* = 5*Y*	5*X* = *Y*
In the basket, the number of apples times five is seven times the number of pears. Use X for the number of apples and Y for the number of pears, then:		In the basket, there are three oranges for every five peaches. Use X for the number of oranges and Y for the number of peaches, then:	
5*X* = 7*Y*	7*X* = 5*Y*	3*X* = 5*Y*	5*X* = 3*Y*

**Table 2:** The likelihood for the evidence X under the condition of hypothesized strategies.

Sentence Structures	Congruent Problems				Incongruent Problems			
Accuracy	Correct		False		Correct		False	
Response Time	Faster	Slower	Faster	Slower	Faster	Slower	Faster	Slower
Automatic Translators	0.91	0.03	0.03	0.03	0.03	0.03	0.91	0.03
Conflict Recognizers	0.91	0.03	0.03	0.03	0.03	0.03	0.03	0.91
Strategic Suppressors	0.91	0.03	0.03	0.03	0.03	0.91	0.03	0.03
Conceptual Integrators	0.91	0.03	0.03	0.03	0.91	0.03	0.03	0.03
Random Guessers	0.25	0.25	0.25	0.25	0.25	0.25	0.25	0.25

**Table 3:** Model fit indices for two hypothesized models.

Model	RMSEA	CFI	TLI	*χ* ^2^	*df*	WRMR
One-factor model	0.161	0.913	0.900	674.210	104	2.337
Two-factor model	0.024	0.998	0.998	116.011	103	0.760

**Table 4:** Means and Standard Deviations of ERs (%) and RTs (ms) for all students (*M* ± *SD*).

Dependent Variable	7th Graders (*n* = 48)	8th Graders (*n* = 50)	11th Graders (*n* = 58)	College Students (*n* = 54)
Error rates				
Congruent	22.06 ± 29.22	15.74 ± 24.96	10.21 ± 21.87	12.15 ± 23.33
Incongruent	78.83 ± 28.32	78.32 ± 30.35	31.07 ± 40.24	27.39 ± 37.05
Response times				
Congruent	6057.95 ± 2604.18	6499.81 ± 2173.67	7886.18 ± 3404.47	6524.75 ± 2135.61
Incongruent	6647.53 ± 4052.34	8858.40 ± 4079.74	10,449.98 ± 2917.36	8277.53 ± 2463.86

**Table 5:** Number of students classified into the hypothesized strategies in every grade.

Strategy	7th Graders (*n* = 49)		8th Graders (*n* = 51)		11th Graders (*n* = 58)		College Students (*n* = 54)	
	*N*	%	*N*	%	*N*	%	*N*	%
Automatic Translators	11	22.45	20	39.22	3	5.17	10	18.52
Conflict Recognizers	9	18.37	10	19.61	8	13.79	2	3.70
Strategic Suppressors	1	2.04	1	1.96	25	43.10	23	42.59
Conceptual Integrators	0	0	1	1.96	5	8.62	3	5.56
Random Guessers	17	34.69	10	19.61	6	10.34	3	5.56
Mixed Strategies Users	11	22.45	9	17.65	11	18.97	13	24.07

**Table 6:** Number of students (%) of different strategy transition profiles across grade levels in the mixed-strategies-users group.

Strategic Profiles	7th Grade	8th Grade	11th Grade	College
Automatic Translator to Conflict Recognizer	2 (15.38)	1 (11.11)	3 (27.27)	0 (0.00)
Cautious Solver to Automatic Translator	7 (53.58)	8 (88.89)	1 (9.09)	0 (0.00)
Strategic Suppressor to Conceptual Integrator	1 (7.69)	0 (0.00)	7 (63.64)	9 (69.23)
Genuinely Idiosyncratic Solver	1 (7.69)	0 (0.00)	0 (0.00)	4 (30.77)

## Data Availability

The data presented in this study are openly available. We have made all our data publicly available on the Open Science Framework at https://osf.io/meetings/apa/ (accessed on 4 April 2026). To facilitate peer review, we provide a private read only link to view our data during the review process here https://osf.io/v3cja/ (accessed on 4 April 2026).

## Notes

1 (0.2 × 0.91)/(0.2 × 0.91 + 0.2 × 0.91 + 0.2 × 0.91 + 0.2 × 0.91 + 0.2 × 0.25) = 0.23.

2 (0.2 × 0.25)/(0.2 × 0.91 + 0.2 × 0.91 + 0.2 × 0.91 + 0.2 × 0.91 + 0.2 × 0.25) = 0.06.

## Appendix A

**Table A1:** Standardized factor loadings and inter-factor correlation for the two-factor CFA model.

Item	Factor Loading	Standard Error	Critical Ratio	*p*-Value
Factor 1 (F1)				
I1	0.939	0.024	39.117	<0.001
I2	0.924	0.027	34.437	<0.001
I3	0.95	0.022	43.977	<0.001
I4	0.942	0.023	40.238	<0.001
I5	0.926	0.026	35.66	<0.001
I6	0.925	0.027	34.575	<0.001
I7	0.954	0.021	44.402	<0.001
I8	0.91	0.03	30.68	<0.001
Factor 2 (F2)				
I9	0.797	0.074	10.766	<0.001
I10	0.783	0.072	10.86	<0.001
I11	0.869	0.052	16.576	<0.001
I12	0.708	0.085	8.344	<0.001
I13	0.897	0.064	13.983	<0.001
I14	0.856	0.069	12.406	<0.001
I15	0.829	0.07	11.824	<0.001
I16	0.772	0.075	10.334	<0.001
Inter-factor correlation				
F1 ↔ F2	−0.291	0.078	−3.728	<0.001

**Table A2:** Number of students classified into the hypothesized strategies in every grade (sensitivity analysis).

Strategy	7th Graders (*n* = 49)		8th Graders (*n* = 51)		11th Graders (*n* = 58)		College Students (*n* = 54)	
	*N*	%	*N*	%	*N*	%	*N*	%
Automatic Translators	11	22.45	20	39.22	3	5.17	10	18.52
Conflict Recognizers	9	18.37	10	19.61	8	13.79	2	3.70
Strategic Suppressors	1	2.04	1	1.96	25	43.10	23	42.59
Conceptual Integrators	0	0	1	1.96	5	8.62	3	5.56
Random Guessers	17	34.69	10	19.61	6	10.34	3	5.56
Mixed Strategies Users	11	22.45	9	17.65	11	18.97	13	24.07

**Table A3:** Means and Standard Deviations of ERs for all students (*M* ± *SD*).

Dependent Variable	7th Graders (*n* = 48)	8th Graders (*n* = 50)	11th Graders (*n* = 58)	College Students (*n* = 54)
Congruent				
Male	17.15 ± 24.74	14.27 ± 24.72	7.31 ± 14.99	8.70 ± 21.30
Female	27.86 ± 31.64	17.33 ± 25.64	17.81 ± 33.43	15.59 ± 25.12
Incongruent				
Male	74.41 ± 30.73	78.54 ± 32.70	25.38 ± 35.19	24.89 ± 38.45
Female	82.58 ± 27.93	78.08 ± 28.28	46.00 ± 49.38	29.89 ± 36.16

**Table A4:** Means and Standard Deviations of RTs for all students (*M* ± *SD*).

Dependent Variable	7th Graders (*n* = 48)	8th Graders (*n* = 50)	11th Graders (*n* = 58)	College Students (*n* = 54)
Congruent				
Male	6596.49 ± 2725.18	6261.55 ± 2156.64	8119.07 ± 2971.25	6411.97 ± 1811.27
Female	5391.18 ± 2338.99	6769.14 ± 2209.18	7234.09 ± 4459.69	6637.54 ± 2448.86
Incongruent				
Male	6608.97 ± 3684.73	9056.01 ± 3398.54	10058.44 ± 2827.30	8742.76 ± 2562.03
Female	7179.34 ± 4355.14	8700.32 ± 4666.43	12059.62 ± 2877.38	7865.98 ± 2346.05
